# Epidemiology and complications of late-onset sepsis: an Italian area-based study

**DOI:** 10.1371/journal.pone.0225407

**Published:** 2019-11-22

**Authors:** Alberto Berardi, Francesca Sforza, Lorenza Baroni, Caterina Spada, Simone Ambretti, Giacomo Biasucci, Serenella Bolognesi, Mariagrazia Capretti, Edoardo Carretto, Matilde Ciccia, Marcello Lanari, Maria Federica Pedna, Vittoria Rizzo, Claudia Venturelli, Crisoula Tzialla, Laura Lucaccioni, Maria Letizia Bacchi Reggiani

**Affiliations:** 1 Unità Operativa di Terapia Intensiva Neonatale, Dipartimento Integrato Materno-Infantile, Azienda Ospedaliero-Universitaria Policlinico, Modena, Italy; 2 Medico in formazione, Scuola di Specializzazione in Pediatria, Università degli Studi di Modena e Reggio, Modena, Italy; 3 Terapia Intensiva Neonatale, Dipartimento Ostetrico e Pediatrico, Istituto di Ricovero e Cura a Carattere Scientifico (IRCCS), Arcispedale Santa Maria Nuova, Reggio Emilia, Italy; 4 Unità Operativa di Microbiologia, Azienda Ospedaliero-Universitaria S. Orsola-Malpighi, Bologna, Italy; 5 Unità Operativa di Pediatria, Ospedale G da Saliceto, Piacenza, Italy; 6 Unità Operativa di Terapia Intensiva Neonatale, Ospedale Infermi, Rimini, Italy; 7 Unità Operativa di Neonatologia, Dipartimento Del Bambino, Della Donna E Delle Malattie Urologiche, Azienda Ospedaliero-Universitaria Sant’Orsola–Malpighi, Bologna, Italy; 8 Laboratorio di Microbiologia, Dipartimento Interaziendale di Diagnostica per Immagini e Medicina di Laboratorio, Istituto di Ricovero e Cura a Carattere Scientifico IRCCS, Arcispedale Santa Maria Nuova, Reggio Emilia, Italy; 9 Unità Operativa di Terapia Intensiva Neonatale, Dipartimento Materno Infantile, Ospedale Maggiore, Bologna, Italy; 10 Unità Operativa di Pediatria, Dipartimento Del Bambino, Della Donna E Delle Malattie Urologiche, Azienda Ospedaliero-Universitaria Sant’Orsola–Malpighi, Bologna, Italy; 11 Unità Operativa di Microbiologia, Laboratorio Unico Ausl della Romagna, Pievesestina Cesena, Italy; 12 Unità Operativa di Terapia Intensiva Neonatale e Pediatrica, Ospedale Civile M. Bufalini, Cesena, Italy; 13 Struttura Complessa di Microbiologia e Virologia, Azienda Ospedaliero-Universitaria Policlinico, Modena, Italy; 14 Neonatologia, Patologia Neonatale e Terapia Intensiva Neonatale, Fondazione IRCCS Policlinico “San Matteo”, Pavia, Italy; 15 Dipartimento di Medicina Specialistica, Diagnostica e Sperimentale, Azienda Ospedaliero-Universitaria S. Orsola-Malpighi—Università di Bologna, Bologna, Italy; Centre Hospitalier Universitaire Vaudois, FRANCE

## Abstract

**Background:**

Most studies regarding late-onset sepsis (LOS) address selected populations (i.e., neonates with low birth weight or extremely preterm neonates). Studying all age groups is more suitable to assess the burden of single pathogens and their clinical relevance.

**Methods:**

This is a retrospective regional study involving paediatric departments and NICUs in Emilia-Romagna (Italy). Regional laboratory databases were searched from 2009 to 2012. Records of infants (aged 4 to 90 days) with a positive blood or cerebrospinal fluid (CSF) culture were retrospectively reviewed and analysed according to acquisition mode (whether hospital- or community-acquired).

**Results:**

During the study period, there were 146,682 live births (LBs), with 296 patients experiencing 331 episodes of LOS (incidence rate: 2.3/1000 LBs). Brain lesions upon discharge from the hospital were found in 12.3% (40/296) of cases, with death occurring in 7.1% (23/296; 0.14/1000 LBs). With respect to full-term neonates, extremely preterm or extremely low birth weight neonates had very high risk of LOS and related mortality (> 100- and > 800-fold higher respectively). Hospital-acquired LOS (n = 209) was significantly associated with very low birth weight, extremely preterm birth, pneumonia, mechanical ventilation, and death (p< 0.01). At multivariate logistic regression analysis, catecholamine support (OR = 3.2), central venous line before LOS (OR = 14.9), and meningitis (OR = 44.7) were associated with brain lesions or death in hospital-acquired LOS (area under the ROC curve 0.81, H-L p = 0.41). Commonly identified pathogens included coagulase-negative staphylococci (CoNS n = 71, 21.4%), *Escherichia coli* (n = 50, 15.1%), *Staphylococcus aureus* (n = 41, 12.4%) and Enterobacteriaceae (n = 41, 12.4%). Group B streptococcus was the predominant cause of meningitis (16 of 38 cases, 42%). Most pathogens were sensitive to first line antibiotics.

**Conclusions:**

This study provides the first Italian data regarding late-onset sepsis (LOS) in all gestational age groups. Compared to full-term neonates, very high rates of LOS and mortality occurred in neonates with a lower birth weight and gestational age. Group B streptococcus was the leading cause of meningitis. Excluding CoNS, the predominant pathogens were *Escherichia coli* and *Staphylococcus aureus*. Neonates with hospital-acquired LOS had a worse outcome. Antibiotic associations, recommended for empirical treatment of hospital- or community-acquired LOS, were adequate.

## Introduction

Sepsis is an ongoing public health problem worldwide and a major cause of morbidity and mortality in newborns, especially in neonates with low birth weight (LBW) or of low gestational age.[1–5] Sepsis can be divided into early-onset sepsis (EOS) or late-onset sepsis (LOS), with LOS occurring in infants aged > 3 days of life.[6] EOS occurs mainly after an ascending infection from the genital tract, whereas LOS is usually hospital or community acquired. Improved survival of extremely premature neonates together with prolonged hospitalization and intensive care, including central venous line, parenteral nutrition, and invasive mechanical ventilation, has resulted in increased rates of LOS. [6,7]

Trends in LOS and changes in antimicrobial susceptibility of pathogens are currently under investigation in many countries [8–12] due to the widespread use of intrapartum antibiotics for preventing group B streptococcus (GBS) EOS and the use of antimicrobials in the postpartum period. However, most studies have been conducted in individual centers [9–11] and some have included both culture-proved and culture-negative (suspected) sepsis or cultures obtained from nonsterile sites (e.g., urinary tract) [13]. Furthermore, the cutoff for LOS has varied (e.g., 48 hours, 72 hours, 6 days) [8–10]. Most studies have addressed selected populations, such as neonates with LBW or extremely preterm neonates [5,14–16]. However, studying all age groups is more suitable to assess the burden of a single pathogen, its clinical relevance, and the effectiveness and appropriateness of empiric antibiotic-prescribing protocols. The present study provides epidemiological data regarding incidence, complications, outcomes, and risk factors for LOS in all gestational age infants.

## Materials and methods

### Study design

This study was carried out in a northern region of Italy (Emilia-Romagna) with a network of active GBS surveillance [17,18]. A screening-based strategy for preventing GBS EOS (according to Centers for Disease Control and Prevention guidelines) [19,20] has been in place in the entire region since 2003 [21,22]. The surveillance network includes 8 microbiological laboratories, 10 level 1 centers (< 1000 live births/year; inborn criteria: ≥ 2000 g, ≥ 35 weeks’ gestation), 4 level 2 centers (> 1000 live births/year; inborn criteria: ≥ 1500 g, ≥ 32 weeks’ gestation), and 8 neonatal intensive care units (NICUs; all with inborn neonates; no restrictions for accepting outborn neonates). Microbiological laboratories were asked to report isolates grown (from January 1, 2009, to December 31, 2012) in blood or cerebrospinal fluid (CSF) cultures from live-born infants (age, 4–90 days) of all gestational ages. Clinicians were asked to complete a standardized form for each case of culture-proved LOS identified by the laboratory. Demographics and clinical data, included infecting organism, clinical symptoms, antimicrobial therapies, days of invasive mechanical ventilation, catecholamine support, and outcome (death or brain lesions), were obtained from admission records by surveillance officers. The study protocol was approved by the Ethical Committee of the coordinating center (Azienda Ospedaliero-Universitaria Policlinico di Modena; Prot. 173/13). The data collected was strictly anonymous. Given the impossibility of retrospectively recovering the consent to all the infants included in the study, the research ethics committee waived the need for the consent

### Clinical and microbiological practices

During the study period, the rate of antenatal steroid use in preterm deliveries was approximately 85%. In all centers, neonates with birth weight < 1000 g were given fluconazole prophylaxis. According to the center policy, a single (1 mL) or double blood culture was obtained before antibiotic treatment in each neonate with clinically suspected LOS. CSF culture was recommended in any case of suspected LOS but was deferred in unstable infants. Blood cultures were processed using automated systems (Bactec 9240; Becton Dickinson, Heidelberg, Germany; Bactalert; bioMérieux, Craponne, France).

Coagulase-negative Staphylococci (CoNS) were included in the list of pathogens causing LOS if infants had clinical signs of sepsis and received intravenous vancomycin or semisynthetic penicillin (e.g., oxacillin, nafcillin) for ≥ 5 days [8,9,23]. Two or more cultures identifying the same CoNS (with the same antimicrobial susceptibility) within 10 days were considered the same episode of LOS [12]. CoNS that did not meet these criteria (n = 244; 75.3%) were considered contaminants.

Micrococci, *Aerococcus* species, *Corynebacterium* species, *Propionibacterium* species, *Bifidobacterium* species, and *Bacillus* species grown in a single culture (n = 25) were also considered contaminants and excluded from analysis.

### Definitions

*Preterm neonates*: neonates born at ≤ 36 ^6/7^ weeks gestation.
*Extremely preterm neonates*: neonates born at 23 to 28 ^6/7^ weeks’ gestation.
*LBW*, *very low birth weight (VLBW)*, and *extremely low birth weight (ELBW)* neonates: birth weight <2500, 1500 and 1000 g, respectively.
*LOS*: growth of pathogens from a normally sterile site (blood and/or CSF) in infants aged 4 to 90 days.[6,10,11,23]
*Pneumonia*: positive blood culture associated with respiratory symptoms and a characteristic radiographic appearance (streaky or confluent lobar opacification that persisted for >24 h).[17]
*Meningitis*: Clinical symptoms associated with a positive result on CSF culture; or a positive result on blood culture and CSF pleocytosis (defined as the presence of 20 white blood cells/mm^3^ and <45,000 red blood cells/mm^3^).[17,24]
*Community- or hospital-acquired LOS*: cases occurring within or after 48 hours from hospital admission.[25–27]
*LOS-related death*: death occurring within 7 days from the positive blood culture or clearly related to complications due to LOS.[10]
*Brain lesion*: Injury detected for clinical indications (i.e. CNS infection or cardiovascular compromise) and confirmed by brain ultrasound or MRI.

### Statistical analyses

Analyses were performed using STATA/SE 14.2 for Windows and MedCalc® version 9.3 (MedCalc Software, Ostend, Belgium).

Continuous variables are expressed as the means ± standard deviations or the medians and ranges. Categorical data are expressed as numbers (percentages). Student’s t-test and Levene’s test for assessing homoscedasticity, the Mann-Whitney rank sum test and χ2 test, or Fisher’s exact test were used to compare, respectively, the continuous and categorical variables between groups.

Brain lesions and death were considered the most severe complications and were studied through uni- and multivariate logistic regression analyses. Invasive mechanical ventilation and catecholamine support were not included in this composite outcome because they are risk factors for developing brain lesions or death and because the decision to use mechanical ventilation or catecholamine support may be subjective.

Several variables were evaluated as possible risk factors for brain lesions or death and they were presented in the univariate analysis; the multivariate logistic regression model was built on the basis of a stepwise selection, with entry criteria = 0.05 and stay criteria = 0.1. There were only a few case fatalities and brain lesions in community-acquired LOS; thus, the multivariate logistic regression analysis was performed only for hospital-acquired LOS.

In order to assess the multicollinearity, the correlation coefficient and VIF (variance inflation factor) were checked. A level of correlation coefficient ≥ 0.9 and a VIF value > 10 were considered critical values. The best subset of predictors in the multivariate models was determined basing on the lowest values of Akaike's information criterion (AIC) and the Bayesian information criterion (BIC).

Analyses were conducted separately for hospital- and community acquired LOS. Because of few case fatalities and brain lesions in community-acquired LOS, the multivariate logistic regression analysis was performed only for hospital-acquired LOS.

The accuracy of the model was verified with the Hosmer-Lemeshow goodness-of-fit test and the area under the ROC curve (AUROC) was also reported. All P-values refer to two-tailed tests of significance. P<0.05 was considered significant, and 0.05<p<0.1 was considered an indication of a trend.

The total number of live births and the numbers of preterm, VLBW and ELBW neonates were provided by the Regional Health Agency and the regional database of the Vermont Oxford Network (VON).

## Results

During the study period, there were 146,682 live births (92.6% full term; 7.4% preterm). The laboratory database identified 624 isolates, 269 of which were discarded as contaminants (CoNS, n = 244; others, n = 25). Fifteen of the remaining 355 isolates were excluded from the final analysis because maternal and/or neonatal data were unavailable; these 15 microorganisms were *Staphylococcus aureus* (n = 3), *Candida parapsilosis* (n = 3), GBS (n = 2), *Acinetobacter* species (n = 1), *Enterococcus faecalis* (n = 1), *Escherichia coli* (n = 1), *Pseudomonas aeruginosa* (n = 1), *Stenotrophomonas maltophilia* (n = 1), *Streptococcus mitis* (n = 1), and *Streptococcus pyogenes* (n = 1).

Complete data sets were available for the remaining 340 microorganisms, which were isolated from blood alone (n = 307), CSF alone (n = 11), or both blood and CSF (n = 22). Nine CoNS were ultimately discarded, as they belonged to the same infective episode. Therefore, the final analysis was performed based on 331 episodes in 296 infants (LOS incidence rate, 2.3/1000 live births), of whom 184 (62.2%) were male and 112 (37.8%) were female. The median birth weight was 2550 g (range, 380–4845 g; IQR, 900–3208 g) and the median gestational age was 36 weeks (range, 22.0–42.0 weeks; IQR, 27.0–39.0 weeks). Approximately half of LOS cases (50.7%) were exposed to IAP and were premature (n = 167; 50.5%) or had LBW (n = 165; 49.8%). Neonates of > 34 weeks’ gestation were more likely than those < 34 weeks to undergo lumbar puncture during LOS (65/183 [35.5%] vs 23/148 [15.5%]; odds ratio [OR], 2.99; 95% CI, 1.7–5.4; *P* = .01). Table 1 shows the cases of LOS and their complications according to their gestational age and birth weight. The highest incidences of LOS, catecholamine support, invasive mechanical ventilation, pneumonia, brain lesions, and mortality occurred in extremely preterm neonates or neonates with extremely LBW. At least 1 episode of LOS occurred in 14.9% of preterm neonates of < 28 weeks’ gestation and in 12.2% of neonates with extremely LBW. With respect to full-term neonates, the risk of LOS (≥ 1 episode) increased approximately 135-fold in extremely preterm neonates or 110-fold in neonates with extremely LBW. The risk of sepsis-related mortality increased approximately 1055-fold in extremely preterm neonates or 815-fold in neonates with extremely LBW.

**Table 1:** Cases of LOS, incidence rates, complications and mortality according to gestational age and birth weight.

	Full-Term neonates	Preterm neonates (<37 weeks gestation)	Extremely preterm neonates (<28 weeks gestation)	VLBW (birth weight <1500 g)	ELBW (birth weight <1000 g)	All
Live births	135,592	11090	569	1840	735	146,682
*Neonates with LOS, n (incidence/1000 LBs)*	150 (1.1)	146 (13.2)	85 (149.4)	112 (60.9)	90 (122.4)	296 (2.0)
*Episodes of LOS, n (incidence/1000 LBs)*	164 (1.2)	167 (15.1)	99 (174.0)	129 (70.1)	102 (138.8)	331 (2.3)
*Case fatalities, n (incidence/1000 LBs)*	3 (0.02)	18 (1.6)	12 (21.1)	15 (8.2)	12 (16.3)	21 (0.1)
*Catecholamine support, n (%)**^a^*	6 (3.7)	37 (22.2)	22 (22.2)	29 (22.5)	25 (24.5)	43 (13.0)
*Mechanical ventilation, n (%)**^a^*	3 (1.8)	66 (39.5)	48 (48.5)	59 (45.7)	51 (50.0)	69 (20.8)
*Pneumonia, n (%)**^a^*	21 (12.8)	81 (48.5)	54 (54.5)	68 (52.7)	57 (55.9)	102 (30.8)
*Brain lesions at hospital discharge, n (%)**^b^*	13 (8.7)	23 (15.8)	18 (21.2)	21 (18.8)	19 (21.1)	36 (12.2)

ELBW, extremely low birth weight; LBs, live births; LOS, late-onset sepsis; VLBW, very low birth weight.

^*a*^ Percentage was calculated based on the number of episodes of LOS.

^*b*^ Percentage was calculated based on the number of neonates with LOS. Brain lesion were confirmed through ultrasound or MRI studies. Lesions include: ventricular enlargement or post-haemorrhagic hydrocephalus (n = 6); white matter damage or periventricular leukomalacia (n = 5); intraventricular haemorrhage (n = 3); subdural empyema or ventriculitis (n = 3); basal ganglia lesion (n = 1); cerebritis (n = 1); impaired cerebellar growth (n = 1); porencephalic cyst (n = 1); unspecified lesions (n = 15).

### Pathogens causing LOS, mode of acquisition and age at presentation

Gram-positive microorganisms represented the majority of the isolates (n = 212; 64.0%); among them, CoNS accounted for 21.4% (n = 71), *Staphylococcus aureus* 12.4% (n = 41), GBS 11.8% (n = 39), *Enterococcus species* 11.2% (n = 37), non-group B streptococci 6.3% (n = 21), and other Gram-positive bacteria 0.9% (n = 3). Gram-negative microorganisms were identified in 105 cases (31.7%); among them, *Escherichia coli* represented 15.1% (n = 50), *Enterobacteriaceae* 12.4% (n = 41), *Pseudomonas species* 2.7% (n = 9), and other genera 1.5% (n = 5). Fungi represented 4.2% of LOS cases (n = 14); among them, *Candida albicans* accounted for 2.4% (n = 8), *Candida parapsilosis* 0.6% (n = 2), and *Candida famata*, *glabrata*, *lusitaniae*, and *tropicalis* 1.2% (n = 1 each). No association was found between IAP exposure and specific pathogens or Gram-positive or Gram-negative pathogens as a group.

**Fig 1:**
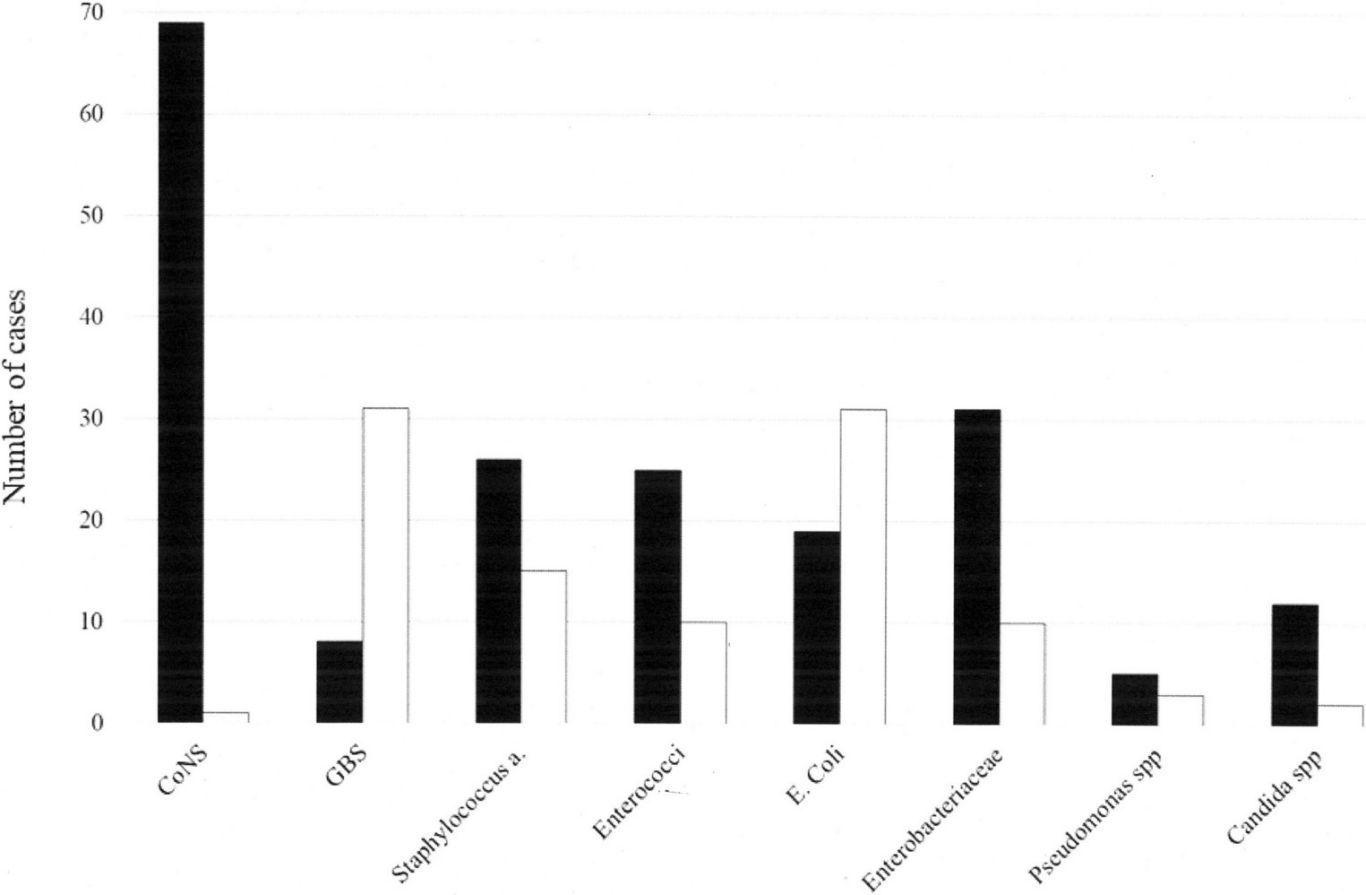
Prominent pathogens in hospital- or community-acquired late-onset sepsis cases. CoNS, coagulase-negative staphylococci; GBS, *group B streptococcus*. One neonate had both community and hospital acquired LOS. Full bars, hospital-acquired LOS. Empty bars, community-acquired LOS. Pathogens causing LOS include: CoNS: *Staphylococcus epidermidis* (n = 52), *S*. *warneri* (n = 7), *S*. *haemolyticus* (n = 2), *S*. *capitis* (n = 5), *S*. *hominis* (n = 1), *S*. *caprae* (n = 1), polymicrobial CoNS (n = 3). Enterococci: *E*. *faecalis* (n = 25), *E*. *faecium* (n = 11), *E*. *avium* (n = 1), Enterobacteriaceae: *Klebsiella pneumoniae* (n = 18), *Klebsiella oxytoca* (n = 5), *Enterobacter aerogenes* (n = 3), *Enterobacter cloacae* (n = 10), *Serratia marcescens* (n = 3), *Pantoea spp* (n = 1), *Proteus mirabilis* (n = 1) *Pseudomonas spp*: *Pseudomonas aeruginosa* (n = 7), *Putida* (n = 1), *Oryzihabitans* (n = 1), *Candida spp*: *Candida albicans* (n = 8), *C*. *tropicalis* (n = 1), *C*. *lusitaniae* (n = 1), *C*. *famata* (n = 1), *C*. *Glabrata* (n = 1), *C*. *parapsilosis* (n = 2).

Hospital-acquired LOS accounted for 63.1% of cases (n = 209; 1.4/1000 live births), while community-acquired LOS accounted for 35.3% (n = 117; 0.79/1000 live births) (date of hospital admission was not reported in 5 cases). The median age at presentation of LOS was 23 days (IQR, 12.0–40.0 days). Symptoms developed significantly earlier in hospital-acquired LOS (median, 21 days; range, 4.0–83.0 days; IQR, 11.75–38.0 days) than in community-acquired LOS (median, 28 days; range, 4.0–90.0 days; IQR, 13.0–49.5 days) (P < .01). Fig 1 shows the prominent pathogens according to the mode of acquisition of LOS. GBS and *Escherichia coli* were predominantly community acquired, whereas the remaining pathogens, namely CoNS, were predominantly hospital acquired.

**Fig 2:**
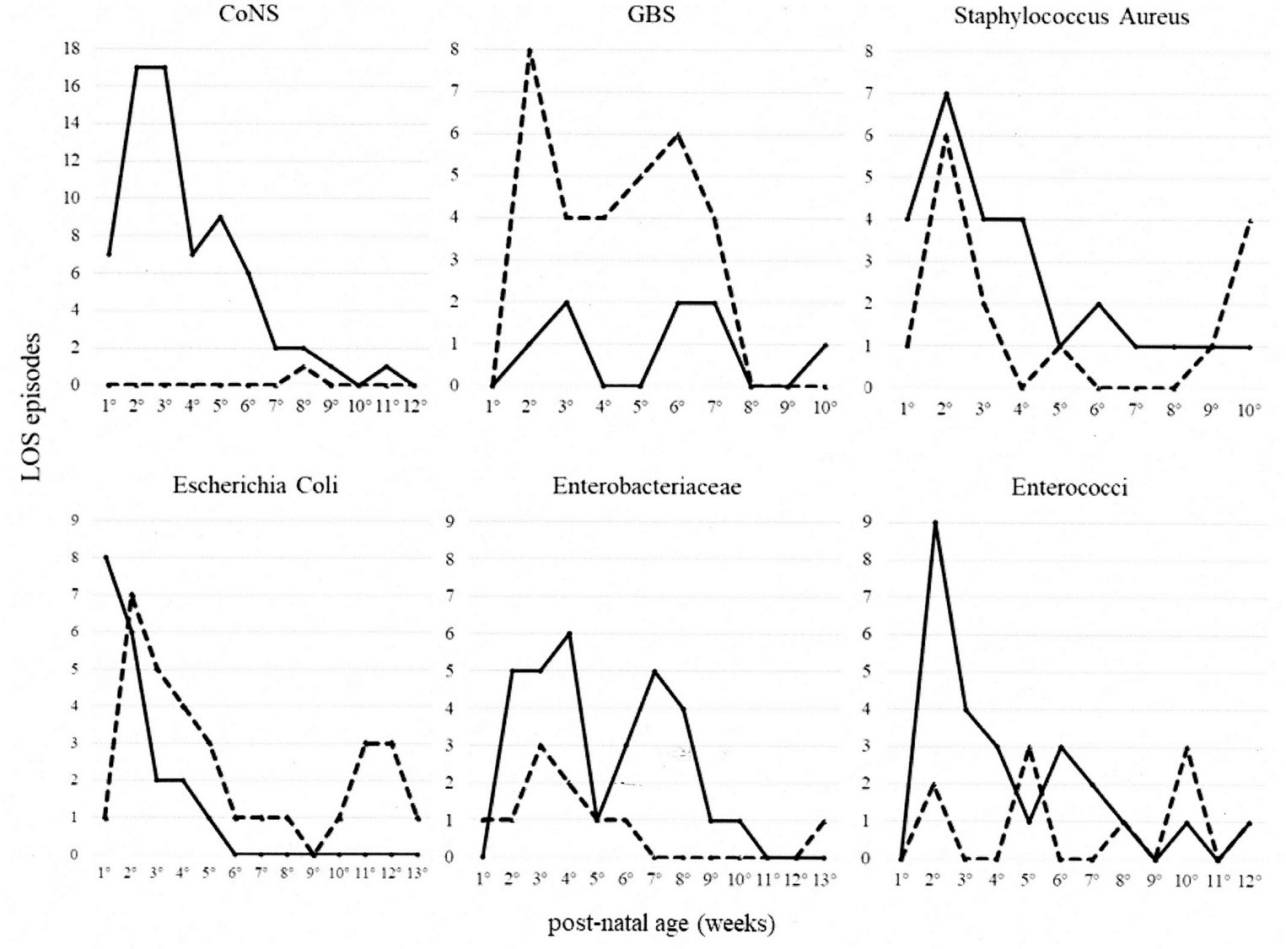
Pathogens according to age at presentation in hospital- and community-acquired LOS. CoNS, coagulase-negative staphylococci; GBS, *group B streptococcus*. Continuous line, hospital-acquired LOS. Dashed line, community-acquired LOS.

The pathogens causing hospital- and community-acquired LOS according to age at LOS confirmation are shown in Fig 2. CoNS were predominantly hospital acquired, with a peak incidence in the first 3 weeks of life; GBS was predominantly community acquired.

Table 2 compares demographics and clinical characteristics of hospital- and community-acquired LOS. Hospital-acquired LOS was associated with higher incidences of pneumonia, mechanical ventilation, catecholamine support, and mortality as well as lower median birth weight, gestational age, and lower rates of gram negative organisms.

**Table 2:** Demographics and clinical characteristics of hospital- and community-acquired late-onset sepsis.

	All episodes n = 326 †	LOS, hospital acquired n = 209	LOS, community acquired n = 117	p
Median birth weight, *g (IQR)*	2485 (900–3208)	1055 (735–2650)	3180 (2890–3500)	< 0.01
Median gestational age, *wks (IQR)*	36 (27–39)	28 (26–36)	39 (38–40)	< 0.01
Median age at the onset of sepsis, *d*	22 (12–40)	21 (12–38)	28 (13–49)	< 0.01
Preterm birth, *n (%)*	165 (50.6)	158 (75.6)	7 (6.0)	<0.01
Preterm birth (< 28 weeks’ gestation), *n (%)*	98 (30.1)	98 (46.9)	0	<0.01
Very low birth weight, *n (%)*	128 (39.3)	128 (61.2)	0	<0.01
Cathecolamine support, *n (%)*	41 (12.6)	37 (17.7)	4 (3.4)	<0.01
Mechanical ventilation, *n (%)*	68 (20.9)	66 (31.6)	2 (1.7)	<0.01
Gram positive pathogens, *n (%)*	208 (63.8)	138 (66.0)	70 (59.8)	0.31
Gram negative pathogens, *n (%)*	104 (31.9)	59 (28.2)	45 (38.5)	<0.01
Fungi, *n (%)*	14 (4.3)	12 (5.7)	2 (1.7)	0.15
Pneumonia, *n (%)*	100 (30.7)	89 (42.6)	11 (9.4)	<0.01
Meningitis, *n (%)* ^§^	38 (43.7)	14 (38.9)	24 (47.1)	0.57
Brain lesions due to sepsis, *n (%)* ^¶^	36 (12.3)	27 (14.7)	9 (8.0)	0.17
Case fatalities, *n (%)* ^¶^	21 (7.2)	20 (10.9)	1 (0.9)	<0.01

d, days; LOS, late-onset sepsis; wks, weeks.

^†^ type of acquisition could not be established in five cases; they were excluded from the total LOS (n = 326 instead of n = 331) and account for minimal inconsistencies (when comparing Tables 1 and 2) of the total number of pneumonia, mechanical ventilation and catecholamine support.

^§^ percent rates of meningitis were calculated on the number of neonates who underwent lumbar puncture (36 in hospital acquired late-onset sepsis, and 51 in community-acquired late-onset sepsis)

^¶^ number and % rates of these outcomes were calculated on the basis of the number of patients (and not the number of episodes).

**Fig 3:**
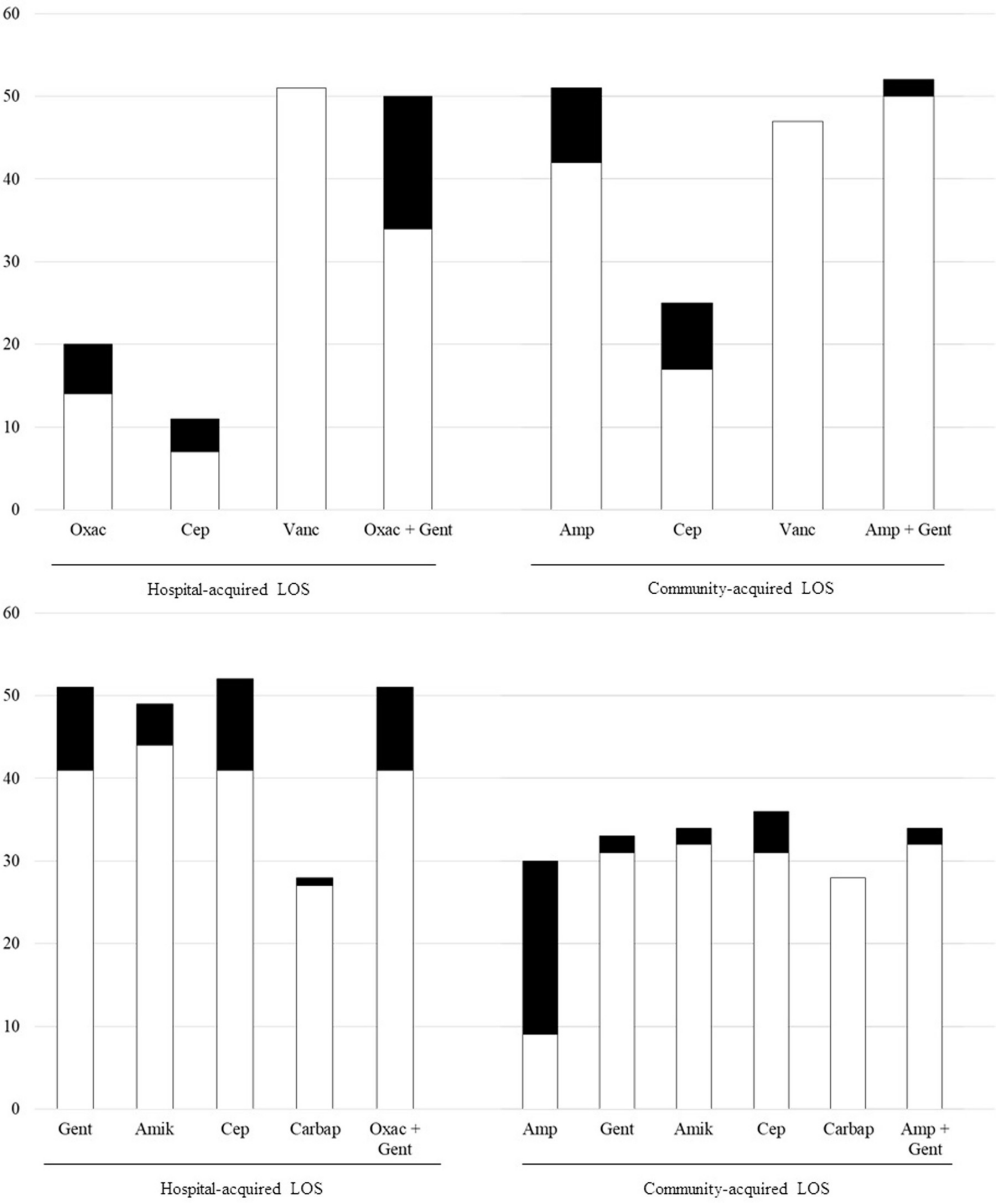
**Sensitivity and resistance of gram positive (panel A) and gram negative (panel B) pathogens as a whole.** Amp, ampicillin; Gent, gentamicin; Cep, third generation cephalosporins; LOS, late-onset sepsis; Oxac, oxacillin; Vanc, vancomycin. White columns, sensitive. Black columns, resistant or intermediate.

Fig 3 reports antimicrobial resistance of gram positive and gram negative pathogens as a whole. Pathogens were mostly sensitive to first line antibiotics or common empirical associations (ampicillin plus an aminoglycoside for community-acquired LOS; an antistafilococcal penicillin plus an aminoglycoside for hospital-acquired LOS)

### Pathogens causing meningitis

There were 38 cases of meningitis among 331 episodes of LOS (11.5%). Among them, 33 had a positive CSF culture (22 with a concomitant positive blood culture) and 5 had CSF pleocytosis and a positive blood culture. Gram-positive microorganisms (n = 30; 78.9%) were responsible for most meningitis cases, with GBS being the predominant pathogen (n = 16; 42.1%), followed by CoNS (n = 5; 13.2%), *Staphylococcus aureus* (n = 3; 7.9%), *Enterococcus species* (n = 3; 7.9%), *Streptococcus pneumoniae* (n = 1; 2.6%), and other streptococci (n = 2; 5.3%). Gram-negative microorganisms accounted for only 7 cases (18.4%): *Escherichia coli* (n = 4; 10.5%) was followed by *Haemophilus influenzae*, *Pseudomonas species*, *and* Enterobacteriaceae (n = 1 each; 7.8%). *Candida albicans* accounted for only 1 case (2.6%).

### Pathogen-related adverse outcomes and risk factors

**Fig 4:**
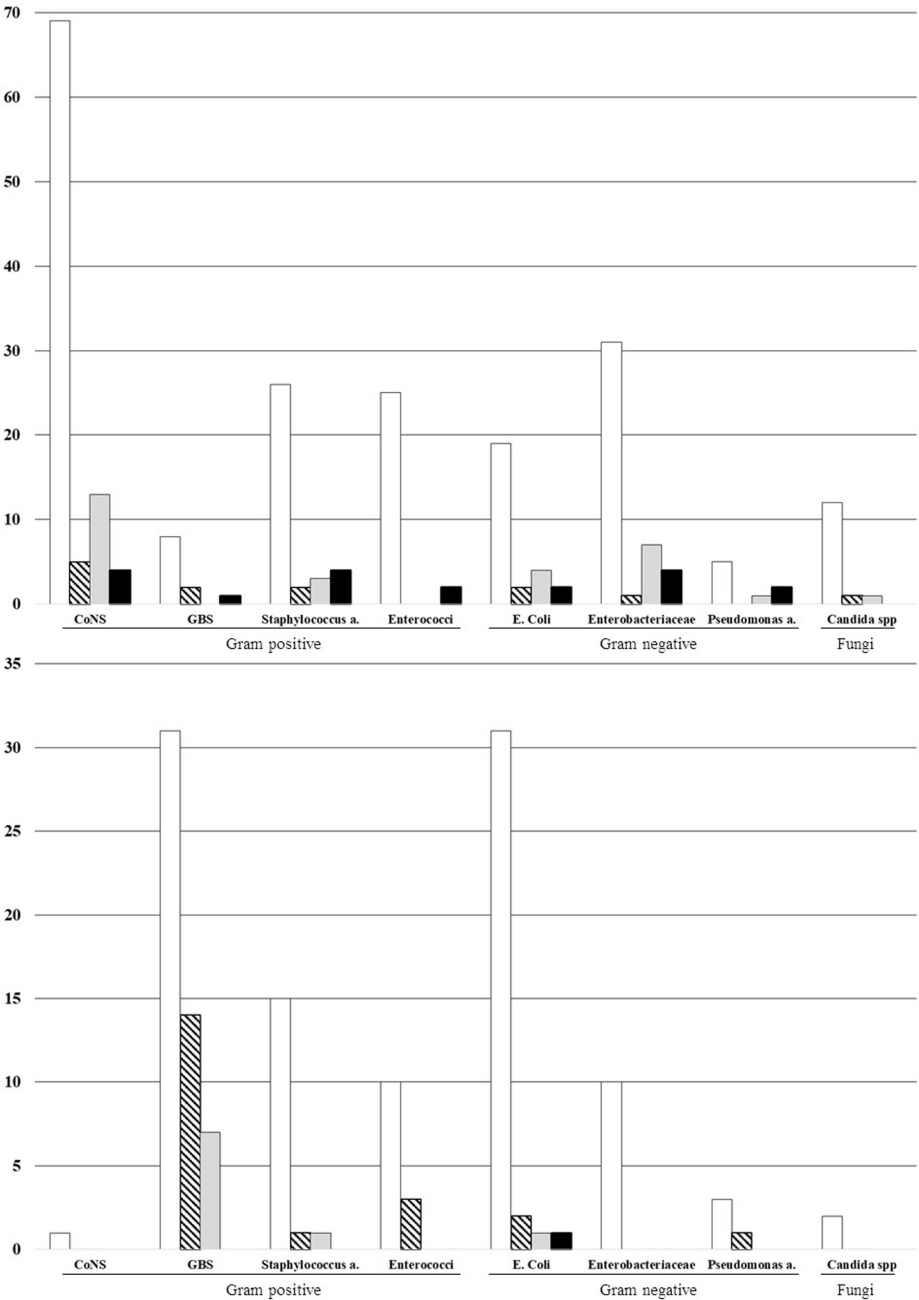
**Number of total cases, meningitis, brain lesions and case fatalities according to each pathogen in hospital- (panel A) and community-acquired LOS (panel B).** CoNS, coagulase-negative staphylococci; GBS, *group B streptococcus*. Empty bars, total cases of late-onset sepsis. Oblique striped bars, cases of meningitis. Grey bars, cases with brain lesions. Black bars, case fatalities.

Thirty-six neonates (12.2%) had brain lesions at hospital discharge and 21 neonates (7.1%) died. Fig 4 shows the total number of cases, meningitis, brain lesions and case fatalities according to each pathogen in hospital- and community-acquired LOS. No fatal cases were due to fungi. Mortality rates in hospital-acquired LOS were comparable among Gram-positive and Gram-negative pathogens. In contrast, case fatalities were rare in community-acquired LOS.

According to the univariate analysis, several variables were associated with death and/or brain lesions at hospital discharge (Table 3). Multivariate logistic regression analysis, performed only for hospital-acquired LOS, showed that catecholamine support (OR, 3.2), central venous line before LOS (OR, 14.9), and meningitis (OR, 44.7) were associated with brain lesions or death (area under the receiver operating characteristics curve, 0.81; *P* = .41; correctly classified, 81.4%). All but 1 case fatality were due to hospital-acquired LOS, whereas most (7/10) cases with brain lesions in community-acquired LOS were due to GBS.

**Table 3:** Uni- and multivariate logistic regression analyses of risk factors for death and/or brain lesions at discharge from hospital in hospital- and community-acquired LOS.

	Hospital-acquired		Hospital-acquired		Community-acquired	
	Univariate analysis		Multivariate Analysis		Univariate analysis	
	OR (95%CI)	P	OR (95%CI)	p	OR (95%CI)	p
*Cathecolamine support*	6.3 (2.8–14.1)	<0.001	3.2 (1.1–9.6)	0.033	12.6 (1.6–101.9)	0.017
*Meningitis*	7.9 (2.3–27.2)	0.001	44.7 (5.1–390)	0.001	57.2 (6.7–486)	<0.001
*Mechanical ventilation during LOS*	2.4 (1.2–4.9)	0.012	**-**	**-**	25.5 (2.1–312)	0.011
*Multi-organ failure*	3.1 (1.1–13.4)	0.032	**-**	**-**	NA	
*Antibiotics before LOS*	2.8 (1.0–7.5)	0.048	-	-	3.4 (0.6–19.3)	0.163
*Central venous line before LOS*	4.0 (0.9–18.0)	0.101	14.9 (1.3–168.2)	0.029	2.7 (0.3–27.3)	0.388
*Very low birth weight*	2.3 (1.1–5.0)	0.029	**-**	**-**	NA	
*Preterm birth 28 weeks’ gestation)*	2.3 (1.2–4.7)	0.016	**-**	**-**	NA	
*Pneumonia*	2.3 (1.2–4.6)	0.015	-	-	5.1 (1.1–23.6)	0.037
*Mechanical ventilation before LOS*	1.9 (0.8–4.4)	0.271	-	-	NA	
*More than 1 episode of LOS*	1.3 (0.5–3.5)	0.555	-	-	21.6 (3.1–151)	0.002
*Prolonged membrane rupture (> 18 hours)*	1.7 (0.8–3.8)	0.183	-	-	3.7 (0.4–39.4)	
*LOS due to CoNS*	1.2 (0.6–2.4)	0.598	-	-	NA	
*No intrapartum antibiotic prophylaxis*	0.5 (0.2–1.0)	0.036	-	-	0.6 (0.2–2.3)	0.477

CoNS, coagulase-negative staphylococci; CSF, cerebrospinal fluid; LOS, late-onset sepsis; MOF, multi-organ failure; NA, not assessable because no neonates with brain lesions underwent mechanical ventilation before LOS, had multiorgan failure, was born preterm, very low birth weight or had LOS due to CONS.

## Discussion

Most existing studies on LOS either have been carried out in single centers [9], focus entirely on preterm infants or those with very LBW [5,14,28], or do not report full epidemiological data according to gestational age [8,9,29]. To our knowledge, only 2 recent European multicenter studies (in Switzerland and Greece) provide data concerning all gestational age neonates; however, their data were obtained from a network of tertiary care centers [26,30].

The current study provides area-based information regarding cases of LOS in an Italian cohort of neonates admitted to NICUs, level 1, or level 2 centers, enabling important comparisons to be made. Indeed, with respect to full-term neonates, the risk of LOS and associated mortality was very high in neonates of lower gestational age or with extremely LBW (> 100- and > 800-fold higher, respectively). To enable comparisons between countries, we reported the number of cases, complications, and age at onset of LOS for each pathogen. The overall incidence of LOS (2.3/1000 live births) was comparable to that found recently in the UK (2.2–3/1000 live births, by excluding CoNS) [8,12] but higher than that found in Switzerland (1.14/1000 live births) [26]. The proportion of neonates with very LBW (39%) was lower than the 60% to 90% [9,10,12] reported in studies focusing on neonates admitted to NICUs. The median gestational age in hospital- and community-acquired LOS (28 and 39 weeks, respectively) was comparable to Switzerland (27 and 39 weeks, respectively), [26] but different from that found in hospital-acquired LOS in Greece (33 weeks) [30].

Even mortality associated with hospital- and community-acquired LOS (11% and 1%, respectively) was very close to that found in Switzerland (12% and 0%, respectively) [26]. Similarly, in the current study, hospital-acquired LOS affected neonates of lower gestational age or with LBW and was more severe than community-acquired LOS, as rates of pneumonia, mechanical ventilation, catecholamine support, and death were significantly higher. Approximately 12% of neonates with extremely LBW and 15% of extremely preterm neonates had ≥ 1 episode of hospital-acquired LOS.

Brain lesions at hospital discharge and mortality were associated with central venous line before LOS and catecholamine support, but the strongest association was found with meningitis. Approximately two-thirds of neonates with meningitis had a sterile blood culture, highlighting the continued need for full sepsis workup (including lumbar puncture) in any case of suspected sepsis. Of note, the rate of meningitis (43.7%) was higher in the current study than in other studies [26], possibly because our rate was calculated based on the number of neonates who actually underwent lumbar puncture.

Gram-positive microorganisms accounted for the majority of LOS cases. CoNS were the most frequent microorganism (accounting for ~ 20% of cases), followed by *Staphylococcus aureus*. The proportion of CoNS varies widely in the literature (20%-54%) but is usually higher than that found in our study [6,8–10,25,31]. Bacteremia with CoNS may reflect a true infection but more likely represents contamination [8,11], and there is debate regarding its inclusion in infection surveillance data. We adopted the criteria provided in other reports [23]. However, it is unclear whether CoNS were always responsible for cases of LOS and related complications, namely, brain lesions at hospital discharge or meningitis. The predominant Gram-negative species was *Escherichia coli*, as has been reported in other studies [12].

The burden of each pathogen changed according to acquisition type (hospital or community). Approximately 65% of LOS cases were hospital acquired (responsible for all but 1 case fatality), and CoNS, *Staphylococcus aureus*, Enterococci, Enterobacteriaceae, and fungi were the predominant isolates. However, 35% of LOS cases were community acquired, with GBS and *Escherichia coli* being the predominant pathogens. GBS had a peak incidence in the first few weeks of life in full-term neonates, but the peak was delayed in preterm newborns. These findings have been previously reported and likely reflect different modes of GBS acquisition in preterm vs full-term neonates [27]. GBS was also the primary pathogen responsible for meningitis, which can lead to long-term disability in approximately half of cases [27]. Finally, the pathogens were mostly sensitive to first-line antibiotics. Thus, common empirical recommendations for treating hospital- or community-acquired LOS were acceptable. However, we expect further reduction of resistance, as an antimicrobial stewardship program was implemented in our region in 2014 to 2015 [32].

This study has several limitations. First, because of its retrospective design, we were unable to retrieve complete information for all cases of LOS, and some data may have been biased. However, > 95% of cases were retrieved. Furthermore, the low rate of lumbar puncture performed may not reflect the actual burden of meningitis, especially among neonates with extremely LBW, who infrequently underwent lumbar puncture. In addition, most neonates underwent cerebral ultrasound to detect lesions, although MRI is more accurate in defining the presence and extent of brain lesions. Thus, lesions may have been more frequent than observed. Although the data collection form explicitly required to report only brain injuries due to LOS, the retrospective nature of the study may have biased results. For example, brain lesions unrelated to LOS (especially in preterm neonates) may have been mistakenly included. Finally, the results obtained in this single region of northern Italy may not be generalizable to the entire country.

In conclusion, LOS had a significant impact on mortality and morbidity in neonates. Compared with full-term neonates, death and brain lesions were greatly increased among neonates of lower gestational age or with LBW, and hospital-acquired LOS was significantly associated with more severe complications and poor outcome. Excluding CoNS, the predominant pathogens were *Escherichia coli* and *Staphylococcus aureus*, whereas GBS caused the majority of meningitis cases. Antibiotic associations for empirical treatment of hospital- or community-acquired LOS were acceptable. Future implications of this study include research concerning trends of resistance after implementing antimicrobial stewardship programs.

## Supporting information

S1 Database (XLS)

S2 Database translated (XLS)
